# Differences in Menstruation-Related Symptoms of University Students Depending on Their Living Status in Japan

**DOI:** 10.3390/healthcare10010131

**Published:** 2022-01-09

**Authors:** Yukie Matsuura, Nam Hoang Tran, Toshiyuki Yasui

**Affiliations:** 1 Department of Reproductive and Menopausal Medicine, Graduate School of Biomedical Sciences, Tokushima University, Tokushima 770-8503, Japan; tosyasui@tokushima-u.ac.jp; 2 Research Center for Higher Education, Tokushima University, Tokushima 770-8502, Japan; tran@tokushima-u.ac.jp

**Keywords:** menstruation-related symptoms, living status, young women, menstrual distress questionnaire

## Abstract

Mothers and family members of young female students play important roles for guiding their self-care strategies for menstruation-related symptoms; which often affect their daily life and academic life. The aim of this study is to clarify the differences in menstruation-related symptoms before and during menstruation in university students living alone and university students living with their family in Japan. We conducted a cross-sectional online survey to assess menstruation-related symptoms before and during menstruation using the menstrual distress questionnaire (MDQ). Among 135 students; the proportion of students living alone was 60.7% and the proportion of students living with their family was 39.3%. Before menstruation; the MDQ total score and the scores for negative affect and behavior change were significantly higher in students living alone than in students living with their family. During menstruation; scores for negative affect and impaired concentration were also significantly higher in students living alone. In addition; before menstruation; scores for an increase in appetite and craving for sweets were significantly higher in students living alone. Thus; living alone affected the psychological aspects of menstruation-related symptoms in young women. The results suggest that university students who live alone should be aware of the importance of talking about their menstruation problems with family members and seeking their advice

## 1. Introduction

Menstruation-related symptoms, including premenstrual symptoms and menstrual pain, have various effects on the daily life of young women. A survey conducted in Japanese female high school students showed that premenstrual symptoms impaired “work efficiency or productivity and home responsibility” in 50.7% of the students, “social life activities” in 23.3% of the students and “relationships with coworkers or family” in 24.0% of the students, and that 11.9% of the students were absent from school for more than 1 day per month due to premenstrual symptoms [1]. A systematic review and meta-analysis of thirty-eight studies in young female school students or university students showed that 20.1% of the students reported an absence from school or university due to dysmenorrhea, and that 40.9% of the students reported classroom performance or concentration being negatively affected [2]. Although 50–80% of women of the reproductive age have at least mild premenstrual symptoms, with 30–40% of women with premenstrual syndrome (PMS), including 3–8% of women with premenstrual dysphoric disorder (PMDD) requiring treatment, most women with premenstrual symptoms repress their symptoms without diagnosis or management [3]. Nevertheless, many young women primarily use self-care for their symptoms and their family plays a part in consultation regarding their symptoms. Mothers, together with school nurses and peers, are the key sources for information on PMS and reproductive health for Japanese high school students [4]. According to a review and meta-analysis of twenty-four studies, the most common source of information about menstrual symptoms other than medical consultations was the family [5]. For Chinese university students, communication about dysmenorrhea was mainly with their mothers (73.4% of the students) and friends (79.0% of the students) [6]. It can be difficult for students to receive support, including information and advice on menstrual symptoms from their family, after starting university and living alone far away from their parents. There have been some studies on the relationship between menstruation-related symptoms and the living status of female university students, but no conclusive results have been obtained. A study conducted in Brazil revealed that menstrual migraine was more frequent in women living together with other students (50%) than in women living alone (16.7%) [7]. In another study conducted in Palestine, no relationship was found between the presence of dysmenorrhea and living status, although students living in dormitories had 1.72 odds of having moderate/severe dysmenorrhea compared to students living with their own families [8]. In Japan, a cohort study conducted on university students at three months after admission to university, showed that students living with their families are more likely to have psychological symptoms during menstruation than students living alone, but no difference was found in the premenstrual symptoms between these two groups of students [9]. Although menstrual cycle-related symptoms include physical, behavioral and emotional symptoms [10], there have been few studies focusing on differences in comprehensive symptoms, especially behavioral symptoms, depending on living status, including living alone or living with the family.

Since 2020, the coronavirus disease 2019 (COVID-19) pandemic has resulted in drastic lifestyle changes, including social distancing and travel restrictions. The pandemic has also resulted in negative changes in the lifestyle of university students, including study, sleep and eating habits, and university students have spent more time in front of screens for online education [11]. The pandemic has also affected menstruation-related symptoms. In Ireland, the pandemic has had negative effects on the reproductive health of women, including worsening premenstrual symptoms (53%) and the appearance of dysmenorrhea (30%) [12]. However, there has been no study on menstruation-related symptoms in relation to living status. Therefore, the aim of this study is to clarify the differences in menstruation-related symptoms before and during menstruation in university students living alone and university students living with their family.

## 2. Materials and Methods

### 2.1. Participants

A cross-sectional online survey was conducted from May to July, in 2021.

The participants were female students majoring in health sciences at a university in Japan. To recruit participants for the study, we conducted an explanation session to a total of 320 students and 169 students agreed to participate.

### 2.2. Procedure

During an online class or face-to-face class, we provided an explanation of the purpose and protocol of the survey and asked students to participate in the survey. The survey was created using the SurveyMonkey^®^ on-line survey tool (https://jp.surveymonkey.com/ accessed on 9 August 2021). We provided the participants the URL and QR code for the survey. On the first page of the web survey, informed consent was obtained by checking the button to agree to participate in the survey, and then the participants continued to answer the questions anonymously. The study was approved by the Ethics Committee of Tokushima University Hospital (approval number: 3932).

### 2.3. Measurements

The questionnaire was developed by the researchers.

#### 2.3.1. Demographic and Lifestyle Exposure Measurements

The questionnaire included questions on demographic factors (grade, age, body weight, body height, age of menarche, currently visiting a gynecologist and receiving hormone therapy); lifestyle (living status, frequencies of eating breakfast and doing exercise, sleeping hours, part-time job, club activities and smoking and drinking habits); rate of online classes and hours spent using smartphone and computers for purposes other than for online classes. We assessed the stress levels of the students for 12 stressors that were selected from previous studies [13,14], including menstrual disorders; physical and health conditions other than menstruation; personality; family relations; relations with friends; romantic relationships; study and academic performance; future prospects; economic conditions; daily life conditions, such as increased housework; part-time jobs and extracurricular activities. The participants rated each item on a 5-point scale from 0 to 4, with 0 indicating no feeling of stress and 4 indicating severe stress.

#### 2.3.2. Menstruation-Related Outcome Measurements

The questionnaire also had questions on present menstrual conditions, including menstrual cycle length and regularity, duration of menstruation and perceived amount of menstrual bleeding, and intensity of menstruation-related symptoms. To assess the degrees of menstruation-related symptoms, we used the menstrual distress questionnaire (MDQ), which is a standard method for assessing cyclical perimenstrual symptoms [15] and also showed Cronbach’s alpha coefficient for reliability [4,16]. The MDQ has been translated into other languages and has been used in many countries, and there is also a Japanese version. The MDQ includes 46 items for self-reported assessment of symptoms and there are 8 scales: 3 somatic scales for pain (6 items), water retention (4 items) and autonomic reactions (4 items); another 3 scales for mood and behavioral changes, namely negative affect (8 items), impaired concentration (8 items) and behavior change (5 items); and two scales for arousal (5 items) and control (6 items) [17]. We asked students to describe their experience of each symptom during each of the 3 stages of the menstrual cycle: before menstruation (4 days before mensuration), during menstruation and the remainder of the cycle. Each symptom was rated from 0 to 4, with 0 indicating no experience of the symptom and 4 indicating severe symptom. In this study, the Cronbach’s alpha coefficient for total MDQ score before and during menstruation was 0.84 and 0.86, respectively. In addition, we added three symptoms about eating habits, including an increase in appetite, craving for sweets and craving for snacks, which were shown to be specific changes before and during menstruation in our previous study [18].

### 2.4. Statistical Analysis

Each categorized variable is expressed as a number with percentage or a mean with standard deviation. We divided the participants into two groups based on their living status, including living alone and living with the family. The significance of differences in variables in the two groups was evaluated by the *t*-test, Fisher’s exact test or the Fisher-Freeman–Halton test. The MDQ score was calculated by the total score and score for each of the 8 scales, and each of them is presented as median (25th percentile, 75th percentile) and mean with standard deviation. The Mann–Whitney U test was used to compare total scores of the MDQ, scores of the eight scales and scores of eating symptoms in the two groups. We conducted additional analyses for each item that was found to have a significant difference according to living status, by using the Mann–Whitney U test. All *p*-values less than 0.05 were considered statistically significant. Statistical analyses were conducted using SPSS Statistics version 28.0 for Windows (IBM Corp., Armonk, NY, USA).

## 3. Results

Of the total of the 320 students who were asked to participate, 169 agreed and responded. Among them, we excluded 18 students who had not completed the MDQ items, 10 students who had received hormone therapy because of gynecological diseases, such as PMS or dysmenorrhea, and 4 students with missing responses to living status. Among the students, 82 students (59.8%) were living alone, 53 students (38.7%) were living with their families, no students were living with friends and 2 students (1.5%) were living in a dormitory. We also excluded the 2 students who were living in a dormitory, and data for 135 students were used for analysis.

### 3.1. Demographic Data

The proportions of students living alone and students living with their family were 60.7% and 39.3%, respectively. There were no significant differences between living status and the grade of students. Body mass index (BMI) for students living alone was significantly higher than that for students living with their family (*p* = 0.036). There were no significant differences between living status and menstrual situations. There was a significant association between living status and frequency of drinking alcohol (*p* = 0.036) (Table 1 and Table 2).

Except for 23 fourth-grade students who were receiving training at hospitals, 74% of the students had taken more than 80% of their classes online. All of the students used smartphones, and the proportions of students who were using smartphones for more than 6 h per day were 22.0% in students living alone and 7.5% in students living with their family. There was no significant difference in the hours of smartphone or personal computer use between the two groups of students, according to living status.

### 3.2. Stress Level

The stress level of daily life, such as increased housework in students living alone was significantly higher than that in students living with their family (*p* < 0.001), but the stress level of family relations tended to be lower in students living alone (*p* = 0.096) (Table 3).

### 3.3. Menstruation-Related Symptoms

Before menstruation, the MDQ total score and scores for negative affect and behavior change were significantly higher in students living alone than in students living with their family. During menstruation, scores for negative affect and impaired concentration were also significantly higher in students living alone than in students living with their family, and the MDQ total score and score for behavior change tended to be higher in students living alone (Table 4).

We conducted additional analyses for each item in the scales of negative affect and behavior change in the premenstrual phase, and negative affect and impaired concentration in the menstrual phase, which were found to be significantly different depending on living status. Before menstruation, the scores were significantly higher in students living alone than in students living with their family for mood swings (*p* = 0.007) and feeling sad or blue (*p* = 0.039) in the negative affect, and for taking naps and staying in bed (*p* = 0.004), staying at home (*p* = 0.002) and avoid social activities (*p* = 0.027) in the behavior change (data was not shown). During menstruation, the scores were significantly higher in students living alone than in students living with their family for mood swings (*p* = 0.023), crying (*p* = 0.013) and restlessness (*p* = 0.01) in the negative affect, and for distractible (*p* = 0.001) in the impaired concentration (data was not shown).

Before menstruation, scores for eating habit symptoms, including an increase in appetite and craving for sweets, were significantly higher in students living alone than in students living with their family (Table 5).

## 4. Discussion

To our knowledge, this is the first study to clarify the differences in menstruation-related symptoms before and during menstruation for university students living alone and university students living with their family. In this study, we found the intensity of menstruation-related symptoms before menstruation and during menstruation was stronger in students living alone than in students living with their family. In addition, the intensities of psychological symptoms, including negative affect and behavior change before and during menstruation and the degree of impaired concentration during menstruation, were stronger in students living alone, but the intensity of the physical symptoms was not significantly different between students living alone and students living with their family.

The intensity of the negative affect was stronger in students living alone than in students living with their family both before and during menstruation. It has been reported that the severity of psychological symptoms during menstruation was stronger in students living with their families than in students living alone, but physical and psychological symptoms were not associated with living status before menstruation [9]. The results of that suggested that, during a period of three months after starting university, students who had left their family home to live alone might be struggling to become independent and to take responsibility for managing their psychological conditions and relieving their symptoms [9]. The results of a survey for students in a different grade might be different. Another study that was conducted in April 2020, showed that the mental health level was not significantly different between either male or female students living alone and students living with someone [19]. It was reported that female students consult with family members, especially their mother, about menstruation and obtain information about menstruation [5,6]; however, it can be difficult for students living alone to receive such support from their family. This might be the reason for differences in menstruation-related symptoms being found according to living status.

Some reports have shown that the COVID-19 pandemic contributed to menstrual distress. In Ireland, the COVID-19 pandemic has had significant effects on the reproductive health of women, including an overall change in their menstrual cycle (46%), worsening premenstrual symptoms (53%) and the new appearance of dysmenorrhea (30%) [12]. In a longitudinal study that was conducted before and during the pandemic for university students in Brazil, the investigation of the “anxiety/stress” symptom of the Premenstrual Symptoms Screening Tool (PSST) revealed that this symptom was more severe before the pandemic, and this can be due to the differences between urban dwellers and people living with families [20]. Another study conducted on 7143 Chinese male and female university students, showed that living with families/parents during the pandemic can be a protective factor against anxiety [21]. In the present study, there was no difference in the intensity of anxiety in the negative affect depending on living status. Anxiety in students might have decreased, given that more than 1 year has passed since the declaration of a pandemic. However, the intensity of mood swings before and during menstruation, the intensity of feeling sad or blue before menstruation and the intensity of crying and restlessness during menstruation were stronger in students living alone than in students living with their family. It was suggested that many Japanese university students living alone live in relatively small apartments, and that their opportunities for in-home exercise and new activities have been limited during the COVID-19 pandemic [19]. The living environment can have effects on the mental health and behavior in students living alone. Regarding behavior, we also found that students living alone had more behavioral changes than students living with their family. Before menstruation, the scores for behavior change, including taking naps, staying in bed, staying at home and avoiding social activities, were higher in students living alone than in students living with their family. A previous study showed that about half of Japanese female university students take naps, stay in bed, stay at home and avoid social activities before menstruation [16], suggesting that young women tend to be less active before menstruation. Another study showed that, regardless of the menstruation period, female university students living with their family tend to feel sleepiness more than students living alone, and they wake up earlier and have less sleeping time than students living alone [22]. This suggests that students with premenstrual symptoms may take more rest and that students who live alone can spend their time freely without being disturbed by others and behavioral changes, such as taking naps, staying in bed, staying at home and avoiding social activities, can occur easily.

It has been reported that living status was not associated with an increased craving for sweets and snacks in Japanese university female students [23]. However, we found that the degrees of increases in appetite and craving for sweets before menstruation were higher in students living alone than in students living with their family. We examined eating habits before and during menstruation, whereas Kasamaki et al. examined eating habits regardless of the menstrual period. This difference can be the reason for the different results regarding eating habits. It is necessary for female students living alone to understand the changes in eating habits during the menstrual period, since students living alone may have an increased craving for sweets and snacks before menstruation. We showed that BMI was higher in students living alone than in students living with their family. A previous study showed that BMI in female university students did not differ depending on living status [22]. Dietary constituents can be involved in the change in BMI because higher consumption of instant food and less consumption of vegetables in students living alone have been reported [22,23]. Students living alone should pay attention to appropriate eating habits.

A study conducted in April 2020 in Japanese university students, revealed that students who lived alone had lower levels of satisfaction with their studies and new activities than students living with someone [19]. We found that stress levels regarding study and academic performance were not different depending on living status. Since our study was conducted more than 1 year after the start of the COVID-19 pandemic, the different survey periods may have been the reason for the difference of results.

We found that the menstruation-related symptoms were more severe in students living alone than students in living with their family. It can be difficult for students living alone to consult with a family member, such as their mother or sister, about menstruation. Students living alone should make efforts to contact their family for advice.

There are several limitations in this study. First, since this study was a cross-sectional survey, a causal relationship during the COVID-19 pandemic could not be clarified. Second, the sample size was small and the participants were selected from only school of health sciences and from only one university. Thus, this study has a limitation regarding the generalizability of the findings. Third, the study might have recall bias by using a self-administered questionnaire. Fourth, we showed data about different types of stresses including menstruation-related stress; therefore, potential interdependence may need a further investigation.

## 5. Conclusions

The present study showed that the Intensity of menstruation-related symptoms, degree of behavior change and intensity of craving for food before menstruation; the intensity of the negative affect before and during menstruation; and the intensity of impaired concentration during menstruation tend to be greater in students living alone than in students living with their family. From these results, we assume that the status of living alone can be related to intensifying some menstruation-related symptoms. However, as this study was cross-sectional study with limited sample size, we may need to conduct further studies with a larger sample size to support our assumption. Moreover, these results can imply that students who live alone need to be aware of the importance of talking about their menstruation problems with a family member, such as their mother or sister, for seeking advice.

## Figures and Tables

**Table 1:** Demographic characteristics and menstrual conditions depending on living status.

Characteristics	Category	Live Alone (*N* = 82)		Live with Family (*N* = 53)		*p*-Value *
		N	%	N	%	
School grade	1st year	42	51.2	18	34.0	0.113
	2nd year	12	14.6	16	30.2	
	3rd year	14	17.1	10	18.9	
	4th year	14	17.1	9	17.0	
Age (years) ^a^		19.4	(1.4)	20.4	(4.7)	0.054
Height (cm) ^a^		157.3	(4.6)	157.4	(5.3)	0.903
Weight (kg) ^a^		52.2	(6.7)	50.3	(5.6)	0.086
BMI (kg/m^2^)^a^		21.1	(2.2)	20.3	(1.9)	0.036
Menarche age (years) ^a^		12.1	(1.5)	12.0	(1.2)	0.574
Menstrual cycle length	24 days or less	0	0.0	2	3.8	0.198
	25–38 days	75	91.5	45	84.9	
	39 days or more	7	8.5	6	11.3	
Menstrual cycle regularity	Regular	41	50.0	22	41.5	0.101
	Sometimes irregular	39	47.6	25	47.2	
	Irregular	2	2.4	6	11.3	
Duration of menstruation	3–7 days	78	95.1	53	100.0	0.154
	8 days or more	4	4.9	0	0.0	
Perceived amount of menstrual bleeding	Light	6	7.3	5	9.4	0.863
	Moderate	63	76.8	41	77.4	
	Heavy	13	15.9	7	13.2	

^a^ mean (SD), * *t*-test, Fisher’s exact test or Fisher–Freeman–Halton exact test.

**Table 2:** Lifestyle characteristics depending on living status.

Characteristics	Category	Live Alone (*N* = 82)		Live with Family (*N* = 53)		*p*-Value *
		N	%	N	%	
Breakfast frequency	Every day	36	43.9	34	64.2	0.142
	5–6 days a week	20	24.4	8	15.1	
	3–4 days a week	14	17.1	6	11.3	
	1–2 days a week	9	11.0	2	3.8	
	None	3	3.7	3	5.7	
Average sleep hours	4 h	2	2.4	1	1.9	0.821
	5 h	15	18.3	8	15.1	
	6 h	35	42.7	19	35.8	
	7 h	27	32.9	23	43.4	
	8 h	3	3.7	2	3.8	
Exercise frequency	Every day	1	1.2	1	1.9	0.304
	5–6 days a week	4	4.9	6	11.3	
	3–4 days a week	6	7.3	7	13.2	
	1–2 days a week	27	32.9	18	34.0	
	None	44	53.7	21	39.6	
Smoking	Yes	0	0.0	1	1.9	0.393
	No	82	100.0	52	98.1	
Drinking	At least once a week	1	1.2	4	7.5	0.036
	Occasionally	22	26.8	7	13.2	
	None	59	72.0	42	79.2	

* Fisher’s exact test or Fisher–Freeman–Halton exact test.

**Table 3:** Intensities of types of stress depending on living status.

Type of Stress	Stress Level	Live Alone (*N* = 82)		Live with Family (*N* = 53)		*p*-Value *
		N	%	N	%	
Menstrual disorders (menstruation and menstruation-related symptoms)	None	18	22.0	12	22.6	0.820
	Mild	19	23.2	16	30.2	
	Moderate	18	22.0	11	20.8	
	Strong	16	19.5	10	18.9	
	Severe	11	13.4	4	7.5	
Physical and health conditions (other than menstruation)	None	37	45.1	27	50.9	0.908
	Mild	28	34.1	18	34.0	
	Moderate	11	13.4	6	11.3	
	Strong	4	4.9	2	3.8	
	Severe	2	2.4	0	0.0	
Personality	None	25	30.5	22	41.5	0.394
	Mild	27	32.9	15	28.3	
	Moderate	21	25.6	10	18.9	
	Strong	4	4.9	5	9.4	
	Severe	5	6.1	1	1.9	
Family relations	None	66	80.5	34	64.2	0.096
	Mild	10	12.2	11	20.8	
	Moderate	5	6.1	4	7.5	
	Strong	1	1.2	4	7.5	
	Severe	0	0.0	0	0.0	
Friend relations	None	46	56.1	34	64.2	0.226
	Mild	27	32.9	13	24.5	
	Moderate	4	4.9	6	11.3	
	Strong	2	2.4	0	0.0	
	Severe	3	3.7	0	0.0	
Romantic relations	None	66	80.5	43	81.1	0.589
	Mild	9	11.0	8	15.1	
	Moderate	4	4.9	2	3.8	
	Strong	3	3.7	0	0.0	
	Severe	0	0.0	0	0.0	
Study and academic performance	None	21	25.6	15	28.3	0.961
	Mild	23	28.0	15	28.3	
	Moderate	24	29.3	16	30.2	
	Strong	10	12.2	6	11.3	
	Severe	4	4.9	1	1.9	
Future prospects	None	23	28.0	13	24.5	0.585
	Mild	18	22.0	16	30.2	
	Moderate	22	26.8	17	32.1	
	Strong	9	11.0	3	5.7	
	Severe	10	12.2	4	7.5	
Economic conditions	None	52	63.4	38	71.7	0.412
	Mild	11	13.4	9	17.0	
	Moderate	13	15.9	5	9.4	
	Strong	4	4.9	0	0.0	
	Severe	2	2.4	1	1.9	
Daily life conditions (increased housework, etc.)	None	32	39.0	42	79.2	<0.001
	Mild	19	23.2	8	15.1	
	Moderate	25	30.5	2	3.8	
	Strong	5	6.1	1	1.9	
	Severe	1	1.2	0	0.0	
Part-time job	None	59	72.0	36	67.9	0.249
	Mild	9	11.0	12	22.6	
	Moderate	7	8.5	4	7.5	
	Strong	6	7.3	1	1.9	
	Severe	1	1.2	0	0.0	
Extracurricular activities	None	67	81.7	38	71.7	0.074
	Mild	10	12.2	13	24.5	
	Moderate	4	4.9	0	0.0	
	Strong	0	0.0	0	0.0	
	Severe	1	1.2	2	3.8	

* Fisher–Freeman–Halton exact test.

**Table 4:** Intensities of Menstrual Distress Questionnaire (MDQ) symptoms in scales depending on living status.

Scales	No. of Items	Before Menstruation							During Menstruation						
		Live Alone (*N* = 82)			Live with Family (*N* = 53)			*p*-Value *	Live Alone (*N* = 82)			Live with Family (*N* = 53)			*p*-Value *
Pain	6	3.0	(1.8,	7.0)	3.0	(1.0,	5.0)	0.244	8.0	(4.0,	12.0)	7.0	(4.0,	11.0)	0.391
Water retention	4	3.0	(1.8,	7.0)	4.0	(2.0,	7.5)	0.470	4.0	(1.0,	6.0)	3.0	(2.0,	6.0)	0.768
Autonomic reaction	4	1.0	(0.0,	2.0)	0.0	(0.0,	2.0)	0.387	2.0	(0.8,	3.3)	1.0	(0.0,	2.0)	0.150
Negative affect	8	6.0	(1.0,	13.0)	4.0	(0.0,	8.5)	0.021	8.0	(2.8,	13.0)	4.0	(1.0,	9.5)	0.014
Impaired concentration	8	2.0	(0.0,	7.0)	1.0	(0.0,	4.0)	0.103	5.0	(1.0,	11.0)	4.0	(0.0,	6.0)	0.025
Behavior change	5	3.0	(0.0,	9.3)	2.0	(0.0,	4.0)	0.014	7.0	(2.0,	12.0)	4.0	(1.0,	8.5)	0.073
Arousal	5	1.5	(0.0,	4.0)	2.0	(0.0,	3.0)	0.563	1.0	(0.0,	4.0)	1.0	(0.0,	3.0)	0.612
Control	6	0.0	(0.0,	1.0)	0.0	(0.0,	1.0)	0.105	0.0	(0.0,	2.0)	0.0	(0.0,	1.0)	0.092
Total MDQ score	46	24.5	(13.8,	50.5)	20.0	(9.0,	30.0)	0.042	38.5	(20.0,	56.3)	29.0	(12.0,	49.0)	0.057

* Mann–Whitney U test; data are presented as medians (25th percentile, 75th percentile).

**Table 5:** Intensities of eating symptoms depending on living status.

Symptoms	Before Menstruation							During Menstruation						
	Live Alone (*N* = 82)			Live with Family (*N* = 53)			*p*-Value *	Live Alone (*N* = 82)			Live with Family (*N* = 53)			*p*-Value *
Increased appetite	3.0	(0.8,	3.0)	1.0	(0.0,	3.0)	0.013	1.0	(0.0,	2.0)	1.0	(0.0,	2.0)	0.858
Craving for sweets	2.0	(1.0,	3.0)	1.0	(0.0,	3.0)	0.013	2.0	(0.8,	3.0)	1.0	(0.0,	3.0)	0.337
Craving for snacks	1.0	(0.0,	2.0)	0.0	(0.0,	1.0)	0.156	0.0	(0.0,	2.0)	0.0	(0.0,	1.0)	0.463

* Mann–Whitney U test; Data are presented as medians (25th percentile, 75th percentile).

## Data Availability

The data presented in this study are not publicly available because of privacy restrictions.
